# Tick-Borne Relapsing Fever, Southern Spain, 2004–2015

**DOI:** 10.3201/eid2212.160870

**Published:** 2016-12

**Authors:** Luis Castilla-Guerra, Jorge Marín-Martín, Miguel Angel Colmenero-Camacho

**Affiliations:** Hospital Universitario Virgen Macarena, Seville, Spain (L. Castilla-Guerra, M.A. Colmenero-Camacho);; Universidad de Sevilla, Seville (L. Castilla-Guerra, M.A. Colmenero-Camacho);; Hospital de la Merced, Seville (J. Marín-Martín)

**Keywords:** Borrelia, meningitis, neurologic complications, relapsing fever, ticks, tick-borne relapsing fever, vector-borne infections, bacteria, soft ticks, Ornithodoros, zoonosis, Spain

**To the Editor:** Surveillance data indicate that tickborne diseases are substantial and increasing global public health problems (*1*). Various pathogens, including viruses, bacteria, protozoa, and helminthes, are transmitted from ticks to vertebrates (*2*). Tick-borne relapsing fever (TBRF) is a zoonosis that is enzootic in many countries (*3*). This illness is caused by >10 *Borrelia* species and is transmitted to humans through the bite of soft ticks of the genus *Ornithodoros* (*3*).

Currently, TBRF is endemic in various foci around the world. However, few TBRF cases are reported in the United States, and in most western European countries, such as Spain, TBRF occurs sporadically, usually after opportunistic infections in persons exposed to ticks (*3*,*4*). Many authors consider TBRF to be underrecognized and underreported (*5*). Although molecular tools such as PCR can dramatically improve diagnosis of this illness, methods used to diagnose TBRF have changed little since the discovery of the spirochete.

To evaluate the prevalence and clinical features of TBRF in a rural area of southern Spain, we retrospectively reviewed clinical data for all patients ≥14 years of age who sought care for TBRF during January 2004–December 2015 at Hospital de la Merced, a county hospital in Seville, Spain. We defined a case of TBRF as detection of spirochetes on thin- or thick-blood smears or in cerebrospinal fluid (CSF) samples by using conventional microscopy after Giemsa or Wright staining (Figure).

**Figure:**
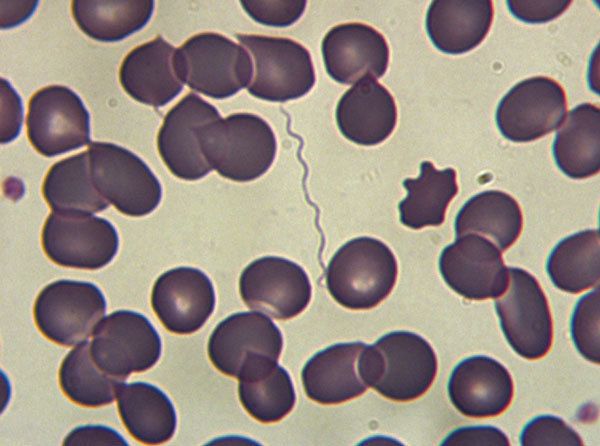
Spirochetes on a thick peripheral blood smear from a patient with tick-borne relapsing fever, southern Spain, 2004–2015. Original magnification ×1,000.

Of 75 patients, 42 (56%) were male and 33 (44%) were female. Mean age was 33 (range 14–72) years. Nine (12%) patients reported tick bites. The most common symptoms were fever (64 [85.3%] patients), headache (41 [54.6%]), vomiting (26 [34.6%]), muscle ache (22 [29.3%]), and abdominal pain (21 [28%]). At the time of the hospital visit, 9 (12%) patients had signs and symptoms suggesting meningeal involvement; 3 (4%) others had clear meningeal signs. These 12 patients underwent lumbar puncture, and CSF abnormalities were found in the 3 (4%) patients with meningismus. Spirochetes were found in the CSF sample of 1 patient. Of the total 75 patients, this patient was the only 1 with spirochetes, and no patient had facial palsy or other neurologic manifestation. The main laboratory findings were elevated C-reactive protein, found in 74 (98.6%) patients; thrombocytopenia, found in 67 (89.3%); and anemia, found in 37 (49.3%).

Preferred treatment was oral doxycycline, which was used for a mean duration of 10 (range 7.3–14) days in 55 patients (73.3%). Among 3 TBRF patients with neurologic involvement, 1 was treated with penicillin G (3 million units/4 h for 10 d), and 2 were treated with ceftriaxone (2 g/d for 14 d). Jarisch-Herxheimer reaction occurred in 7 (9.3%) patients, none of whom had meningitis. All patients recovered completely.

Currently, TBRF is widely distributed in various foci around the world. In much of sub-Saharan Africa, TBRF is associated with a high number of illnesses and deaths. Indeed, it is reportedly the most common bacterial infection from Senegal and is listed among the 10 leading causes of death in children <5 years of age in Tanzania (*6*). Elsewhere in the world, this infection is regarded as rare. Although TBRF borreliosis occurs infrequently in developed countries, our study highlights TBRF endemicity in an area of southern Spain.

Reports on TBRF in Spain are scarce. The only previous study involving numerous cases of TBRF in Spain (*7*) described 230 cases and was published in the early 20th century. That research showed that, although disease caused by *B. hispanica* is less severe than that of other TBRFs, ≈5% of patients had neurologic complications. In our study, 3 (4%) patients had meningitis caused by TBRF borreliosis, a finding that accords with the previous report. 

A recent study conducted among children in southern Spain (*8*) identified 9 cases of TBRF during a 10-year period. Two children, 3 and 5 years of age, had meningeal involvement but no other neurologic complication. Similar to observations in our study, Jarisch-Herxheimer reaction was infrequent, occurring in only 1 of the 9 children.

Neurologic complications are well known features of infection with 2 spirochetes, *B. burgdorferi* and *Treponema pallidum* (*9*). Nevertheless, little is published and known about the predilection of TBRF borreliosis to infect the nervous system. One of the few studies reviewing neurologic involvement of TBRF borreliosis reported that *B. turicatae* and *B. duttonii*, the agents of TBRF in southwestern North America and sub-Saharan Africa, respectively, cause neurologic involvement as often as *B. burgdorferi* causes Lyme disease (*10*).

Our study confirms that TBRF is an endemic, underreported disease in many countries and is common in southern Spain. Although the disease caused by *B. hispanica* is among the less severe illnesses caused by the relapsing fever group, serious neurologic complications can occur. With increasing globalization, physicians will likely see increased numbers of travel-related infections and will face imported and emerging TBRF cases. 
